# The value of NLRP, PCT, and HBP for sepsis-associated acute kidney injury and the effect of anti-inflammatory drugs

**DOI:** 10.1097/MD.0000000000047654

**Published:** 2026-03-13

**Authors:** Zhidong Fang, Shuqin Cui, Xian Liu, Min Shi, Wei Zheng, Jing Xue, Li Chen

**Affiliations:** a Department of Critical Care Medicine, Wu’an First People’s Hospital, Handan City, Hebei Province, China; b Internal Three Departments, Tangshan Third Hospital, Tangshan City, Hebei Province, China; c Department of Critical Care Medicine, North China Petroleum Administration General Hospital, Cangzhou City, Hebei Province, China; d Department of Internal Medicine, Meilanju Community Health Service Station, Baoding City, Hebei Province, China.

**Keywords:** anti-inflammatory drugs, heparin-binding protein, nucleotide-binding oligomerization domain-like receptor protein, procalcitonin, sepsis-associated acute kidney injury

## Abstract

Sepsis-associated acute kidney injury (SA-AKI) is one of the most common and severe complications in clinical practice, with high incidence and mortality rates. This work aimed to demonstrate the predictive value of the inflammatory factors nucleotide-binding oligomerization domain-like receptor protein (NLRP), procalcitonin (PCT), and heparin-binding protein (HBP) for SA-AKI and assess the impact of anti-inflammatory drug therapy on its clinical efficacy. About 120 patients with SA-AKI admitted to our hospital from January 2020 to December 2022 were enrolled, and they were categorized into survival group (82 cases) and death group (38 cases) regarding the outcomes. The levels of NLRP, PCT, and HBP in the blood of both groups were measured, and receiver operating characteristic curves were plotted to evaluate the predictive value of mortality risk factors in SA-AKI patients. The SA-AKI patients received anti-inflammatory drug therapy, and clinical indicators were compared pre- and post-treatment. NLRP, PCT, and HBP in survival group were notably inferior to death group (*P* < .05). The survival group had greatly inferior acute physiology and chronic health evaluation II scores and sequential organ failure assessment scores to death group (*P* < .05). NLRP, PCT, and HBP showed positive predictive values for the prognosis of SA-AKI. After anti-inflammatory drug treatment, patients exhibited reduced levels of inflammation, improved renal function, increased urine output, rapid decline in serum creatinine levels, and milder renal injury (*P* < .05). The anti-inflammatory treatment exhibited a significantly higher overall efficacy rate in patients compared to those for whom the treatment was ineffective (*P* < .05). NLRP, PCT, and HBP, as inflammatory factors, have high predictive value for the early diagnosis and evaluation of SA-AKI. Anti-inflammatory drug therapy effectively reduces inflammation levels, improves renal function, and mitigates kidney damage in patients.

## 1. Introduction

Sepsis is a life-threatening disease caused by severe infection, closely associated with dysregulated immune response in the body, and may lead to organ dysfunction and high mortality rates.^[1]^ During the course of sepsis, patients may develop sepsis-associated acute kidney injury (SA-AKI), a severe complication that adversely affects patient outcomes.^[2]^ The pathogenesis of SA-AKI is complex and multifaceted, involving the release of inflammatory cytokines, endothelial dysfunction, vasodilation, and coagulation disturbances, among others.^[3]^ Approximately 11% to 13% of hospitalized patients and over 50% of intensive care unit patients are reported to suffer from SA-AKI.^[4,5]^ AKI manifests itself as rapid decline in glomerular filtration rate, leading to the impaired excretion of toxins, metabolic waste products, and electrolytes from the body.^[6]^ AKI can be caused by various factors, with common reasons including hemorrhage, hypotension, drug or toxin-induced injury, infections, renal ischemia-reperfusion injury, and urinary tract obstruction, among others.^[7,8]^ Additionally, severe conditions such as sepsis, congestive heart failure, liver dysfunction, and trauma can also trigger AKI.^[9]^ Understanding its pathogenesis and risk factors, early diagnosis, and proactive treatment are critical in preventing disease progression and improving patient outcomes.

In clinical practice, early diagnosis of SA-AKI typically involves assessing the concentration of serum creatinine (SCr).^[10]^ Nevertheless, SCr is not the most optimal clinical indicator for predicting SA-AKI. Renal function is governed by the glomerular filtration followed by tubular reabsorption, which represents the smallest functional unit of the kidney. In the context of sepsis, oliguria is a common occurrence in patients.^[11]^ Some studies have shown that the decrease in neutrophil gelatinase-associated lipocalin (NGAL) in serum serves as an early warning sign for the progression of chronic kidney disease in SA-AKI patients.^[12]^ While healthy individuals typically have low levels of serum NGAL,^[13]^ its concentration can be influenced by various factors, making it less specific for disease diagnosis and susceptible to multiple influences, including the patient’s inflammatory status and current medication use. Although NGAL levels may increase in early AKI, there can be exceptions, thus necessitating longer monitoring and continuous measurements for its use in early SA-AKI diagnosis. Other research has proposed the utility of serum cystatin C (Cys-C), kidney injury molecule-1, NGAL, and fibroblast growth factor-23 (FGF-23) as additional early predictors for SA-AKI.^[14]^ Currently, there is an urgent need for improved AKI predictive biomarkers to enhance the prognosis of septic patients. Understanding the mechanisms underlying SA-AKI can assist in better prevention and management of this severe disease.

In recent years, some biomarkers such as insulin-like growth factor-binding protein 7, tissue inhibitor of metalloproteinases 2, and kidney injury molecule-1 in urine have been extensively studied as potential indicators for early diagnosis of SA-AKI.^[15,16]^ This work investigated the predictive value of nucleotide-binding oligomerization domain-like receptor protein (NLRP), procalcitonin (PCT), and heparin-binding protein (HBP) in SA-AKI, as nucleotide-binding oligomerization domain-like receptors (NLRs) are closely associated with the inflammatory process.^[17,18]^ NLRP in the cytoplasm plays a role as a negative regulatory factor to inhibit inflammation by targeting components of the nuclear factor-κB (NF-κB) signaling to mitigate inflammation.^[19]^ The primary mechanism involves NLRP’s activation and its interaction with tumor necrosis factor receptor-associated factor 6 (TRAF6). In the NF-κB pathway, in its non-activated state, NF-κB is bound to inhibitor IκB. NLRX1, on the other hand, appears to form a complex with cytoplasmic TRAF6. Upon activation, IκB kinase phosphorylates IκB, leading to its degradation and subsequent release of NF-κB, allowing it to translocate into the nucleus and participate in transcriptional regulation. Nevertheless, in the presence of lipopolysaccharides associated with toll-like receptor 4 (TLR4) activation, NLRP and TRAF6 undergo K63-linked polyubiquitination, leading to complex dissociation. Once dissociated, the leucine-rich repeat domain of NLRX1 binds to the kinase domain of the activated IκB kinase complex, thereby inhibiting the activity of the NF-κB pathway.^[20]^ NLRP plays a crucial regulatory role primarily in the mitochondria and cytoplasm, participating in the formation and regulation of inflammasomes, which control the inflammatory response (IR) process. PCT is a peptide hormone synthesized by thyroid C cells, and its levels are often correlated with the severity of infectious diseases.^[21]^ In a retrospective observational study, a total of 753 sepsis patients (including 405 AKI patients) were enrolled for diagnostic monitoring, and the results demonstrated that PCT can serve as a biomarker to predict AKI in sepsis patients. The elevation of PCT is not only correlated with the degree of IR but also serves as a predictive marker for assessing whether patients with systemic infections have AKI.^[22]^ Hence, monitoring serum PCT levels can be an important indicator to evaluate the risk and progression of SA-AKI. HBP is a pro-inflammatory protein released by neutrophils and plays a significant role in mediating inflammation.^[23]^ Studies noted that increased plasma HBP levels are associated with the occurrence of AKI induced by sepsis. HBP is involved in the regulation and maintenance of IRs, leading to an increase in interleukin (IL)-6 production in renal tubular epithelial cells, affecting mechanisms such as vascular permeability and coagulation function, ultimately contributing to the development and progression of AKI.^[24,25]^ Hence, this work aimed to investigate the predictive value of NLRP, PCT, and HBP for SA-AKI.

The release of inflammatory cytokines is a crucial link in pathogenesis of sepsis and SA-AKI, and therapeutically, inhibiting the IR may help alleviate renal damage.^[26]^ In clinical practice, the treatment of SA-AKI is typically comprehensive, involving fluid resuscitation, antibiotic therapy, and the use of vasopressor agents, among others. Currently, some anti-inflammatory drugs and immunomodulators are taken during treatment to reduce the release of inflammatory cytokines and the overall IR. Additionally, anti-inflammatory drugs are widely employed in SA-AKI treatment to control inflammation and mitigate renal injury. Commonly utilized anti-inflammatory drugs include nonsteroidal anti-inflammatory drugs (NSAIDs) such as ibuprofen and glucocorticoid drugs like dexamethasone. These drugs exert anti-inflammatory effects by modulating the production and release of inflammatory mediators, thereby improving the prognosis of SA-AKI to some extent. Rhizoma Coptidis (RC), known as Huanglian in traditional Chinese medicine, possesses anti-inflammatory and antioxidant properties. Studies have found that the extract of RC (RCE) has a protective effect against SA-AKI.^[27]^ Ulinastatin is a natural anti-inflammatory substance that inhibits pancreatic trypsin. Research has shown that ulinastatin can reduce tumor necrosis factor-alpha (TNF-α), IL-6, IFN-γ, while increasing the levels of the anti-inflammatory cytokine interleukin-10 (IL-10).^[28]^ In this work, ulinastatin was utilized as an anti-inflammatory drug to treat sepsis patients to explore its application efficacy in SA-AKI.

In summary, understanding the pathogenesis and related factors of SA-AKI is of great importance in guiding its prevention and treatment. Molecules such as NLRPs, PCT, and HBP play essential roles and are being extensively studied for their involvement and regulatory mechanisms in SA-AKI. Additionally, anti-inflammatory drugs utilized in clinical practice have provided some assistance in the treatment of SA-AKI. Nevertheless, further research is required to gain a deeper understanding of the mechanisms of action of these molecules and their potential therapeutic applications in SA-AKI.

## 2. Materials and methods

### 2.1. Research object

This study was approved by the Ethics Committee of Wu’an First People’s Hospital. This work enrolled 120 patients diagnosed with SA-AKI admitted to our hospital from January 2020 to December 2022 as research subjects. Among them, patients were categorized into survival group and death group regarding the outcomes, including 82 and 38 patients, respectively.

Inclusion criteria: patients aged 18 years or older; clinically diagnosed with sepsis and accompanied by AKI; patients meeting the criteria for kidney injury, that is, sustained increase in SCr levels (>0.3 mg/dL or a 1.5-fold increase from baseline) or urine output < 0.5 mL/kg/h for more than 6 hours; patients voluntarily participating in the study and providing informed consent.

Exclusion criteria: patients with renal insufficiency, such as chronic kidney disease or kidney transplant; patients who previously underwent kidney surgery; patients with severe cardiovascular disease, liver dysfunction, immune system disorders, or other significant infectious diseases; pregnant or lactating female patients; patients unable to provide complete clinical data.

### 2.2. Therapeutic methodologies

Clinical data from 120 patients were collected and organized, including age, gender, heart rate (HR), mean arterial pressure, and medical history. After enrollment, before administering anti-inflammatory drugs, 5 mL of fasting venous blood was collected from all patients in the early morning. The blood samples were immediately centrifuged (3200 *r*/15 minutes), and the supernatant was collected and stored at −80°C for subsequent unified testing.

All patient groups received standardized treatment, including early fluid resuscitation, antimicrobial therapy, vasoconstrictors, mechanical ventilation, and comprehensive management, with bedside blood purification therapy as needed. During the treatment period, all patients received anti-inflammatory drug therapy with ulinastatin. The administration method involved dissolving 2 mL of ulinastatin (National Medical Products Administration Approval Number H20040476; Manufacturer: Guangdong Tianpu Biochemical Pharmaceutical Co., Ltd.; Specification: 2 mL: 1,00,000 units) in 10 mL of 0.9% normal saline injection. The solution was slowly administered intravenously, with 3 injections per day, continuously utilized for 7 days.

Survival group criteria: patients who achieved cure or showed improvement in their condition, with disappearance of disease symptoms and restoration of normal renal function, were classified as cured. Patients who experienced partial relief of disease symptoms, partial recovery of renal function, and some improvement in clinical signs were considered to have shown improvement.

Death group included: clinically deceased patients and those who opted to discontinue treatment due to severe organ failure; patients who were declared deceased when both respiration and heartbeat had ceased; patients exhibited severe and unimproved disease symptoms, worsening of renal function, or even organ failure, leading to the decision to discontinue treatment.

### 2.3. Clinical index observation

Observations included recording the total urine output of all patients pre- and posttreatment for 24 hours. Acute physiology and chronic health evaluation II (APACHE-II) scores and sequential organ failure assessment (SOFA) scores were calculated for all patients pre- and post-treatment.

Biochemical markers were measured using the Indiko Plus fully automated biochemical analyzer (Thermo) to determine the levels of SCr, beta-2 microglobulin (β2-MG), blood urea nitrogen (BUN), and Cys-C in the patients’ blood samples.

Inflammatory cytokine levels were measured using enzyme-linked immunosorbent assay to detect inflammatory cytokines and predictive biomarkers in the patients’ blood. Specifically, TNF-α, interleukin-1 beta (IL-1β), IL-6, and IL-10 were detected, along with the contents of the predictive biomarkers NLRP, PCT, and HBP. Methodology was as follows. Sample collection and pretreatment were performed, followed by adding the test substance solution to the wells of a 96-well plate coated with solid-phase material, allowing the substance to adsorb or covalently bind to the surface. Subsequently, a blocking agent was applied to cover the unbound surface, preventing the generation of nonspecific background signals. The samples were applied to the wells of the solid-phase material after adsorption, allowing the test substance to specifically bind to the antibodies or antigens immobilized on the solid phase. Multiple washes were performed to remove nonspecifically bound substances. Subsequently, enzyme-labeled antibodies or antigens specifically binding to the test substance were applied, forming a sandwich immunoassay. Incubation and washing were continued to remove unbound enzyme-labeled molecules. Next, substrate solution was added, leading to a measurable signal upon reaction with the enzyme label. Finally, reaction was stopped with the stop solution, and the absorbance was measured using equipment such as a spectrophotometer. The presence of the test substance was determined based on the absorbance values, following the instructions strictly according to the relevant guidelines.

A comparative analysis of the therapeutic outcomes following administration of anti-inflammatory medication in patients was conducted, taking into account parameters such as APACHE-II scores, SOFA sepsis scores, levels of inflammatory markers TNF-α, IL-1β, IL-6, and IL-10, diagnostic indicators NLRP, PCT, and HBP, as well as renal functional parameters.

Receiver operating characteristic (ROC) curve analysis was employed to assess the predictive value of NLRP, PCT, and HBP in determining risk factors for mortality in SA-AKI patients.


$$\begin{array}{l} TPR=TP/\left(TP+FN\right) \end{array}$$ (1)

Note: TPR is the true positive rate, TP represents the number of true positive cases, that is, the number of samples that are actually positive cases and correctly predicted as positive cases, and FN represents the number of false negatives, that is, the number of samples that are actually positive cases but are wrongly predicted as negative cases.


$$\begin{array}{l} FPR=FP/\left(FP+TN\right) \end{array}$$ (2)

Note: FPR is a false positive rate.


$$\begin{array}{l} TNR=TN/\left(TN+FP\right) \end{array}$$ (3)

Note: TNR is the true negative rate, FP represents the number of false positives, that is, the number of samples that are actually negative but wrongly predicted as positive cases, and TN represents the number of true negatives, that is, the number of samples that are actually negative cases and are correctly predicted as negative cases.


$$\begin{array}{l} FNR=FN/\left(FN+TP\right) \end{array}$$ (4)

Note: FNR is a false negative rate.


$$\begin{array}{l} AUC=\int \left(TPR\left(FPR\right)\right)dFPR \end{array}$$ (5)

Note: Area under the curve (AUC) is the area under the curve.


$$\begin{array}{l} BMI=Weight\left(kg\right)/Height{\left(m\right)}^{2} \end{array}$$ (6)

$$\begin{array}{lll} & Mortality\;rate\; & =\left(number\;of\;deaths/total\;number\;of\;people\right)\times 100\% \end{array}$$ (7)

### 2.4. Data statistics

The collected clinical data of the patients were organized and analyzed using SPSS 24.0. For metric data that met the normal distribution, results were denoted as mean ± standard deviation (*X̄* ± *s*), and the differences pre- and post-treatment were analyzed using *t*-tests. For metric data that did not meet normal distribution, the results were expressed as median and interquartile range (M [IQR]), and non-parametric tests were utilized for analysis. Count data were presented as percentages (%), and differences were compared using chi-square test. The relationship between sepsis and AKI risk factors was analyzed using binary logistic regression analysis. Pearson correlation analysis was employed to examine correlation between NLRP, PCT, and HBP and the kidney disease: improving global outcomes staging of SA-AKI patients. ROC curves were utilized to analyze predictive value of NLRP, PCT, and HBP for SA-AKI diagnosis, and the sensitivity and specificity of the indicators were evaluated. The AUC represented the diagnostic efficacy. A binary logistic regression analysis was utilized for multi-indicator joint analysis, with a significance level set at *P* < .05.

## 3. Results

### 3.1. Clinical data

**Figure 1.**
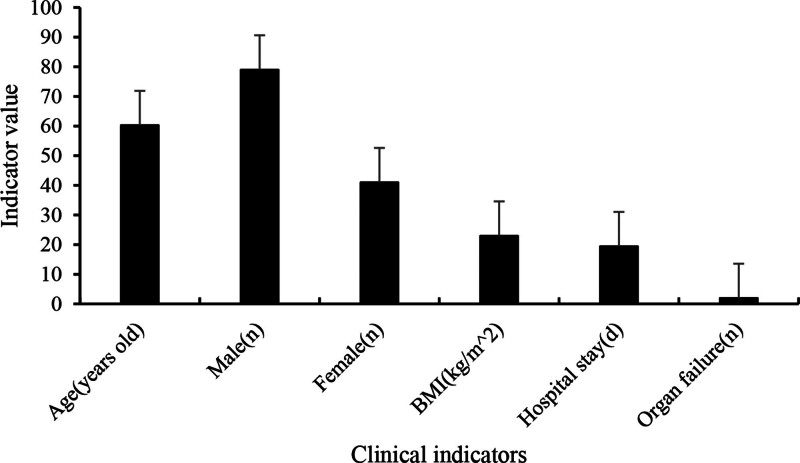
Clinical indicators of patients.

**Figure 2.**
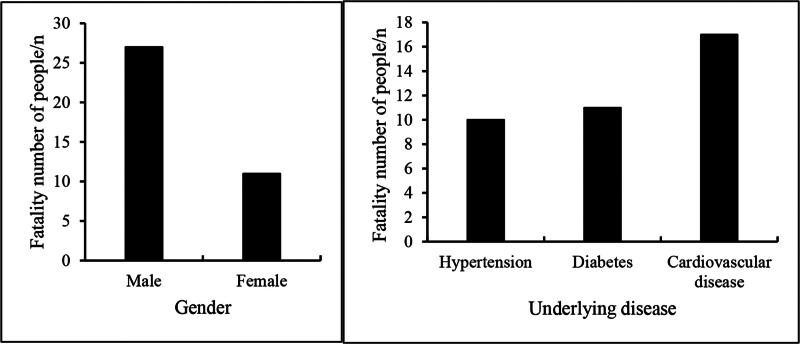
Statistics of the number of deaths among patients with basic diseases.

**Figure 3.**
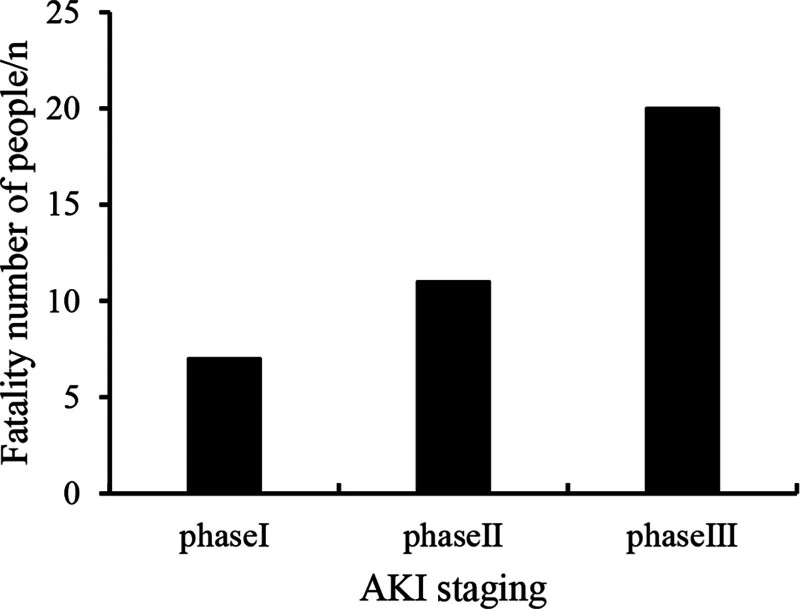
Statistics of patient deaths in AKI staging. AKI = acute kidney injury.

**Figure 4.**
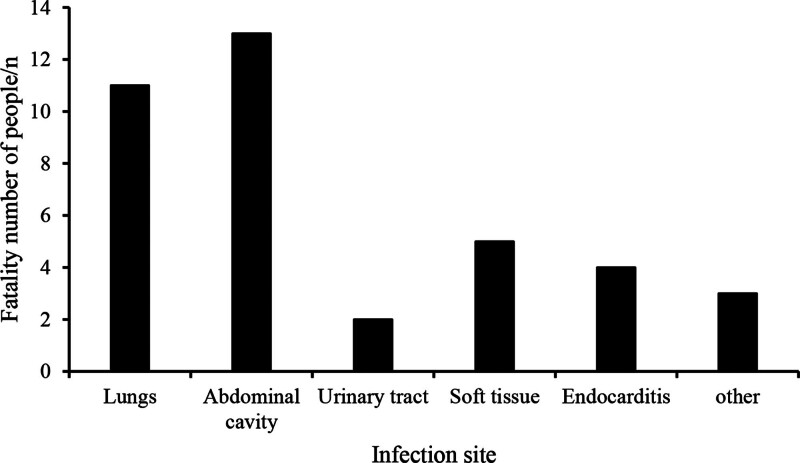
Statistics of the number of deaths at the site of infection in patients.

A total of 120 SA-AKI patients were included in the study, comprising 79 male patients, of whom 27 died, and 41 female patients, of whom 11 died. The average age of the patients was (60.26 ± 9.73) years, with an average body mass index of (22.96 ± 4.62) kg/m^2^. The mean hospitalization duration was (19.42 ± 7.58) days, and the number of organ failures per patient was (1.94 ± 0.68). The clinical information of the patients is presented in Tables 1–3 and Figures 1–4.

**Table 1:** Patients with basic diseases.

Group	Sex		Basic diseases		
	Male	Female	Hypertension	Diabetes	Cardiovascular disease
Case (%)	79 (65.83)	41 (34.17)	43 (35.83)	37 (30.83)	40 (33.33)
Mortality rate (%)	34.18	26.83	23.26	29.73	42.50

**Table 2:** Number of patients with acute renal injury in different stages.

Group	AKI staging		
	I	II	III
Case (%)	30 (25.00)	35 (29.17)	55 (45.83)
Mortality rate (%)	23.33	31.43	36.36

AKI = acute kidney injury.

**Table 3:** Number of people infected with various parts.

Group	Infection site					
	Lung	Abdominal cavity	Urinary tract	Skin and soft tissue	Endocarditis	Others
Case (%)	34 (28.33)	41 (34.17)	7 (5.83)	12 (10.00)	14 (11.67)	12 (10.00)
Mortality rate (%)	32.35	31.71	28.57	41.67	28.57	25.00

### 3.2. Observation of clinical indicators

**Figure 5.**
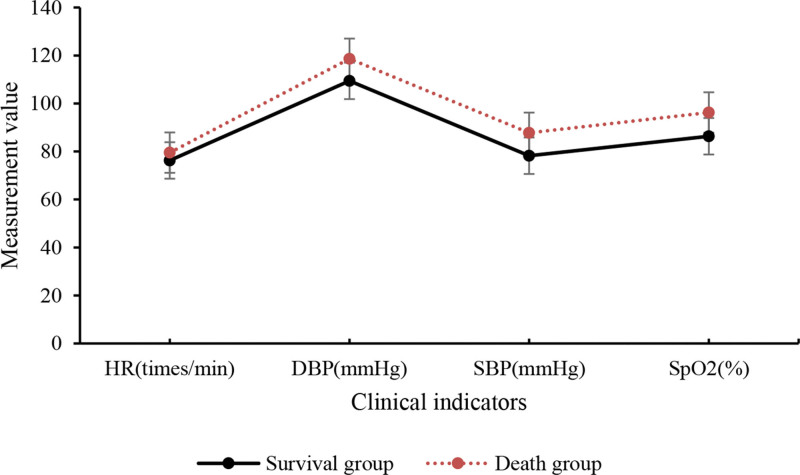
Comparison of clinical indicators.

The clinical indicators of 120 patients were collected and analyzed, and the results are presented in Table 4 and Figure 5. When compared with death group, survival group of SA-AKI patients showed slightly lower values in HR, diastolic blood pressure, systolic blood pressure, and blood oxygen saturation (*P* > .05).

**Table 4:** Comparison of clinical indicators.

Group	Heart rate (beats/min)	Diastolic blood pressure (mm Hg)	Systolic blood pressure (mm Hg)	Blood oxygen saturation (%)
Survival group	76.26 ± 19.37	109.42 ± 36.51	78.23 ± 19.62	86.34 ± 26.53
Death group	79.52 ± 20.43	118.63 ± 39.62	87.79 ± 21.64	96.25 ± 29.74
*t*	0.467	0.628	0.273	0.836
*P*	>.05	>.05	>.05	>.05

**Figure 6.**
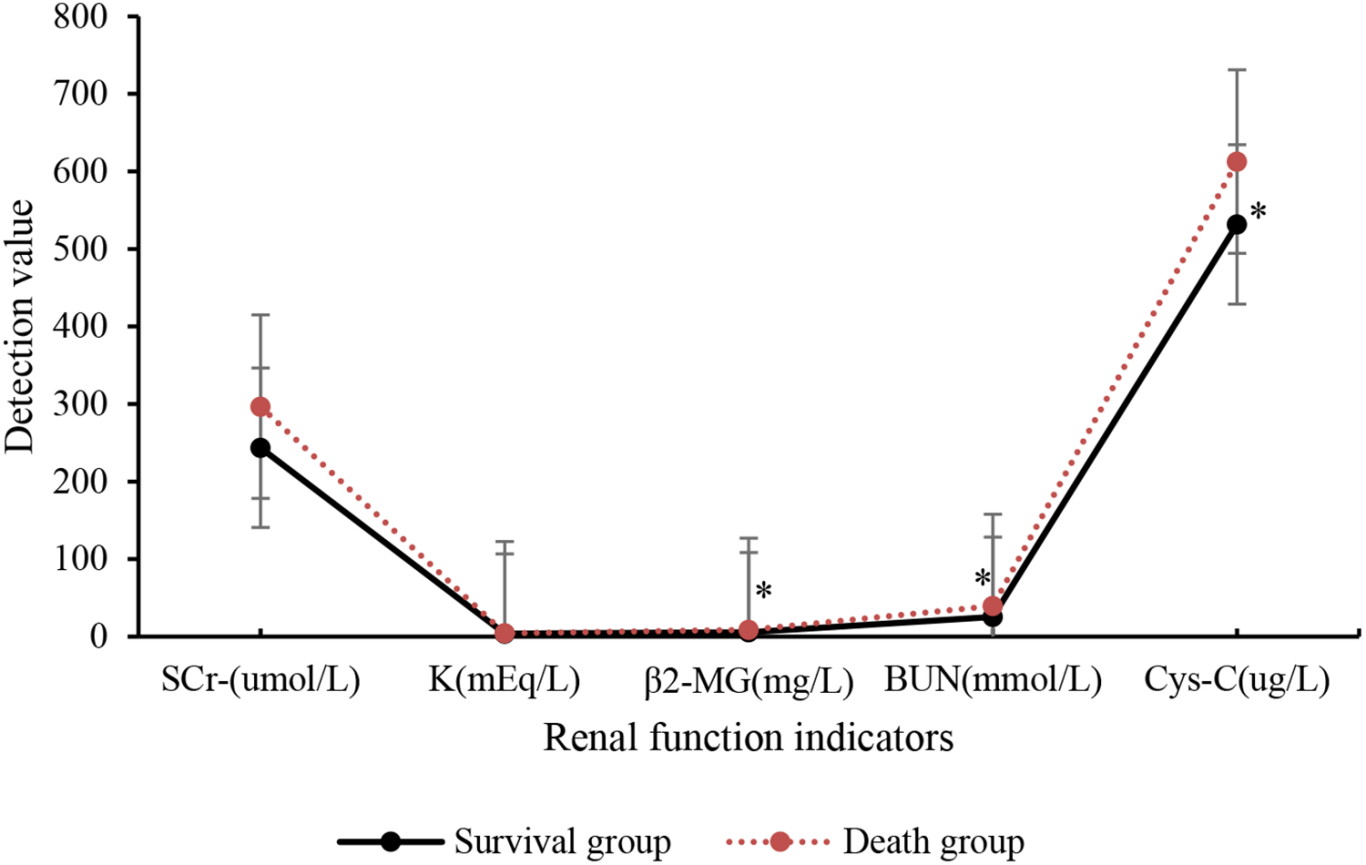
Comparison of renal function indicators. *P < .05 versus death group.

The comparison of renal function indicators between the 2 groups is presented in Table 5 and Figure 6. Survival group of SA-AKI patients had inferior levels of SCr and blood potassium to death group, but not considerable (*P* > .05). Nevertheless, the levels of serum β2-MG and BUN were drastically lower, and the Cys-C levels were notably superior in survival group to death group (*P* < .05).

**Table 5:** Comparison of renal function indicators.

Group	SCr (µmol/L)	Blood potassium (mEq/L)	β2-MG (mg/L)	BUN (mmol/L)	Cystatin C (µg/L)
Survival group	243.61 ± 123.16	3.87 ± 1.16	5.58 ± 1.73*	25.52 ± 7.61*	612.73 ± 162.74*
Death group	296.63 ± 143.73	4.31 ± 1.14	8.69 ± 2.16	39.53 ± 10.42	531.62 ± 132.86
*t*	−1.139	0.294	3.322	4.263	4.524
*P*	>.05	>.05	<.05	<.05	<.05

BUN = blood urea nitrogen, SCr = serum creatinine, β2-MG = beta-2 microglobulin.

* *P* < .05 versus death group.

### 3.3. The level of patient diagnostic markers

**Figure 7.**
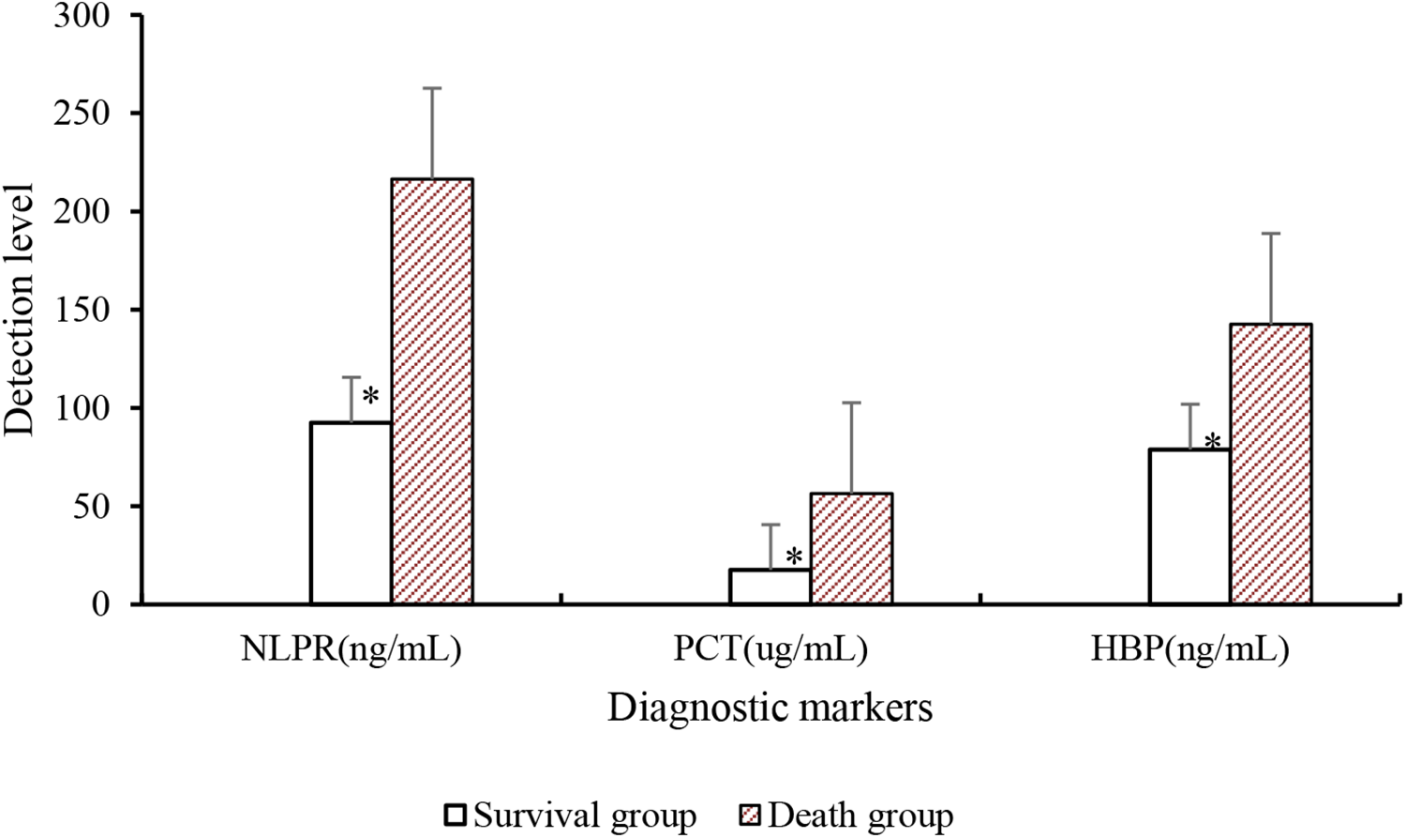
Comparison of the levels of diagnostic markers. *P < .05 versus death group. HBP = heparin-binding protein, NLPR = nucleotide-binding oligomerization domain-like receptor protein, PCT = procalcitonin.

The results are presented in Table 6 and Figure 7. Relative to death group, serum levels of NLRP, PCT, and HBP in survival group of SA-AKI patients were greatly reduced (*P* < .05).

**Table 6:** Comparison of diagnostic biomarker levels.

Group	NLPR (ng/mL)	PCT (µg/mL)	HBP (ng/mL)
Survival group	92.52 ± 21.73*	17.56 ± 7.25*	78.84 ± 20.53*
Death group	216.42 ± 75.62	56.42 ± 18.63	142.53 ± 47.84
*t*	8.323	5.734	7.362
*P*	<.05	<.05	<.05

HBP = heparin-binding protein, NLRP = nucleotide-binding oligomerization domain-like receptor protein, PCT = procalcitonin.

* *P* < .05 versus death group.

### 3.4. Patient APACHE-II score and SOFA sepsis score

**Figure 8.**
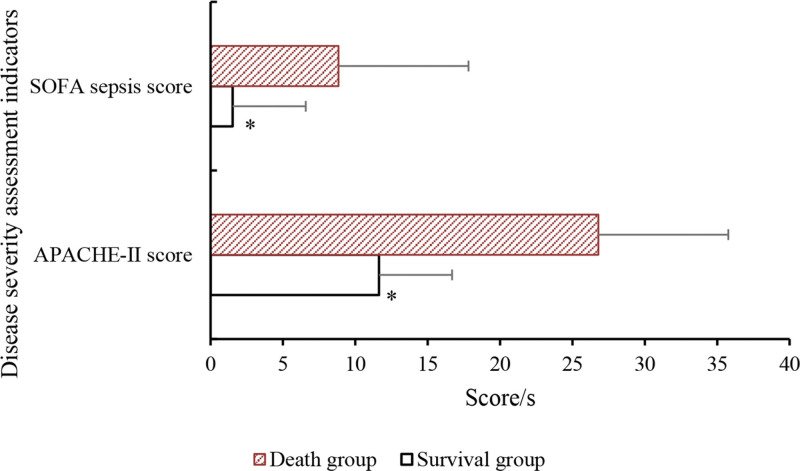
Comparison of APACHE-II score and SOFA sepsis score. *P < .05 versus death group. APACHE-II = acute physiology and chronic health evaluation II, SOFA = sequential organ failure assessment.

The results are presented in Table 7 and Figure 8. The APACHE-II score and SOFA sepsis score of SA-AKI patients in survival group were markedly reduced versus death group (*P* < .05).

**Table 7:** Comparison of APACHE-II score and SOFA sepsis score.

Group	APACHE-II score	SOFA sepsis score
Survival group	11.63 ± 2.74*	1.52 ± 0.47*
Death group	26.79 ± 5.38	8.84 ± 2.51
*t*	2.965	4.734
*P*	<.05	<.05

APACHE-II = acute physiology and chronic health evaluation II, SOFA = sequential organ failure assessment.

* *P* < .05 versus death group.

### 3.5. Effectiveness analysis of NLRP, PCT, and HBP in diagnosing the severity of SA-AKI

**Figure 9.**
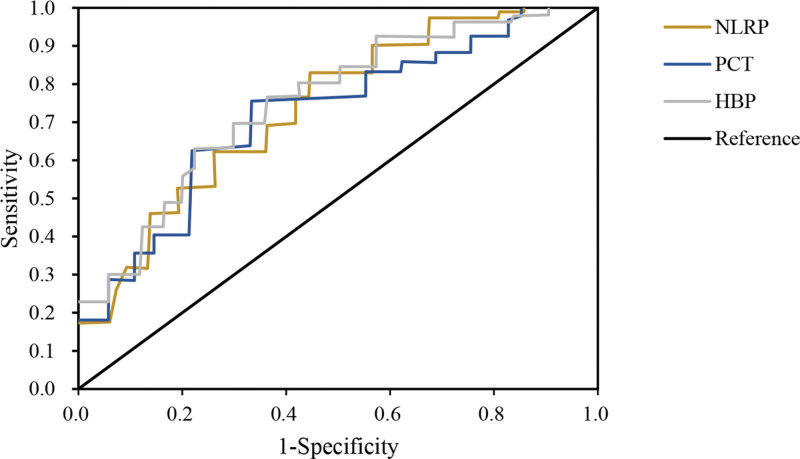
ROCs of NLRP, PCT, and HBP to SA-AKI. HBP = heparin-binding protein, NLRP = nucleotide-binding oligomerization domain-like receptor protein, PCT = procalcitonin, ROC = receiver operating characteristic, SA-AKI = sepsis-associated acute kidney injury.

ROC was drawn to analyze predictive value of NLRP, PCT, HBP and SA-AKI diagnosis (Fig. 9). The results demonstrated that the AUG under the curve of NLRP, PCT and HBP was 0.783, 0.847, and 0.803, respectively, and the difference was substantial (*P* < .05; Table 8).

**Table 8:** Analysis of diagnostic value of NLPR, PCT, and HBP for SA-AKI.

Index	AUC	*P*	Younden’s index	Sensitivity (%)	Specificity (%)
NLRP	0.783	<.05	0.532	82.63	84.51
PCT	0.847	<.05	0.683	73.58	92.63
HBP	0.803	<.05	0.657	80.27	84.62

NLRP = nucleotide-binding oligomerization domain-like receptor protein, PCT = procalcitonin, SA-AKI = sepsis-associated acute kidney injury.

### 3.6. Binary logistic regression analysis for predicting factors related to the severity of SA-AKI

Using serum NLRP, PCT, and HBP levels as covariates, a multiple factor analysis was conducted to assess their predictive correlation with the severity of SA-AKI, as presented in Table 9. The results demonstrated that serum NLRP level (*P* = .009, OR = 1.013), serum PCT level (*P* = .009, OR = 1.013), and serum HBP level (*P* = .009, OR = 1.013) were independent risk factors for the occurrence of SA-AKI.

**Table 9:** Binary logistic regression analysis of factors related to severity of SA-AKI.

Index	β	SE	OR	Wals	*P*	95% CI
NLRP	0.025	0.019	1.013	9.864	.009	0.008–0.058
PCT	0.392	0.146	1.532	9.372	.003	1.213–2.198
HBP	0.038	0.028	1.036	9.971	.009	1.013–0.071

CI = confidence interval, HBP = heparin-binding protein, NLRP = nucleotide-binding oligomerization domain-like receptor protein, OR = odds ratio, PCT = procalcitonin, SA-AKI = sepsis-associated acute kidney injury, SE = standard error.

### 3.7. APACHE-II score and SOFA sepsis score pre- and post-treatment

**Figure 10.**
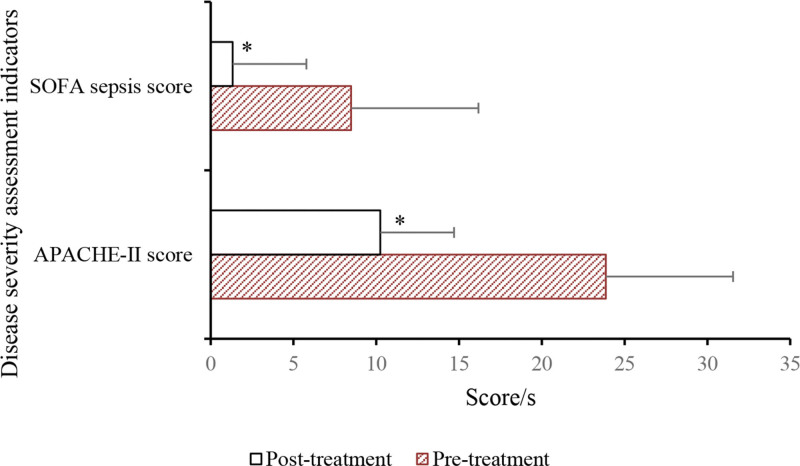
Comparison of APACHE-II score and SOFA sepsis score. *P < .05 versus pretreatment. APACHE-II = acute physiology and chronic health evaluation II, SOFA = sequential organ failure assessment.

APACHE-II scores and SOFA sepsis scores of patients before and after anti-inflammatory treatment are presented as follows. The results demonstrated a remarkable decrease in APACHE-II scores in SA-AKI patients after anti-inflammatory treatment ([23.86 ± 5.36] vs [10.24 ± 2.73], *P* < .05). Similarly, there was a drastic reduction in SOFA sepsis scores after treatment ([8.48 ± 2.61] vs [1.32 ± 0.48], *P* < .05; Fig. 10).

### 3.8. Inflammatory factor levels pre- and post-treatment

**Figure 11.**
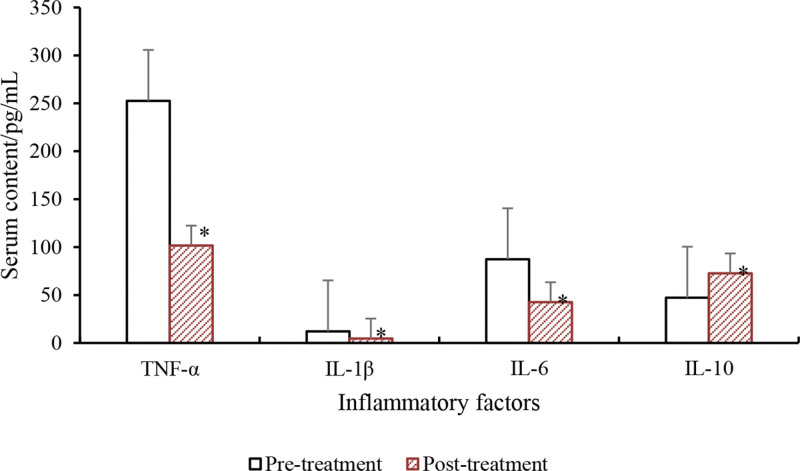
Comparison of inflammatory factor levels. *P < .05 versus pretreatment. IL-1β = interleukin-1 beta, IL-6 = interleukin-6, IL-10 = interleukin-10, TNF-α = tumor necrosis factor-alpha.

Comparison of the inflammatory cytokine levels before and after anti-inflammatory treatment in SA-AKI patients is presented in Table 10 and Figure 11. The results demonstrated a prominent reduction in TNF-α, IL-1β, and IL-6 levels in SA-AKI patients after anti-inflammatory treatment versus pretreatment (*P* < .05). Furthermore, the IL-10 level in the serum of SA-AKI patients was notably elevated after treatment versus pretreatment (*P* < .05).

**Table 10:** Comparison of inflammatory factors.

Group	TNF-α (pg/mL)	IL-1β (pg/mL)	IL-6 (pg/mL)	IL-10 (pg/mL)
Pretreatment	252.51 ± 47.52	12.15 ± 5.52	87.32 ± 23.74	47.26 ± 13.58
Posttreatment	101.63 ± 36.73*	4.68 ± 1.57*	42.61 ± 18.53*	72.63 ± 17.62*
*t*	6.372	4.734	5.273	5.034
*P*	<.05	<.05	<.05	<.05

IL-1β = interleukin-1 beta, IL-6 = interleukin-6, IL-10 = interleukin-10, TNF-α = tumor necrosis factor-alpha.

* *P* < .05 versus pretreatment.

### 3.9. Levels of diagnostic markers pre- and post-treatment

**Figure 12.**
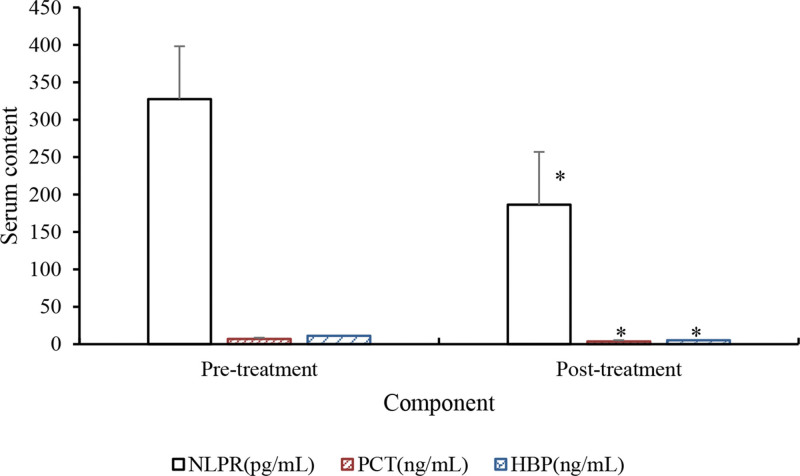
Levels of diagnostic markers in patients. *P < .05 versus pretreatment. HBP = heparin-binding protein, NLRP = nucleotide-binding oligomerization domain-like receptor protein, PCT = procalcitonin.

Comparison of diagnostic biomarker levels before and after anti-inflammatory treatment in SA-AKI patients is presented in Table 11 and Figure 12. The results demonstrated a drastic reduction in NLRP, PCT, and HBP levels in SA-AKI patients after anti-inflammatory treatment versus pretreatment (*P* < .05).

**Table 11:** Levels of diagnostic markers in patients.

Group	NLPR (pg/mL)	PCT (ng/mL)	HBP (ng/mL)
Pretreatment	327.63 ± 46.59	7.02 ± 2.56	11.27 ± 2.53
Posttreatment	186.42 ± 36.73*	3.71 ± 1.92*	5.29 ± 1.26*
*t*	5.628	4.392	4.785
*P*	<.05	<.05	<.05

HBP = heparin-binding protein, NLRP = nucleotide-binding oligomerization domain-like receptor protein, PCT = procalcitonin.

* *P* < .05 versus pretreatment.

### 3.10. Renal function indicators pre- and post-treatment

**Figure 13.**
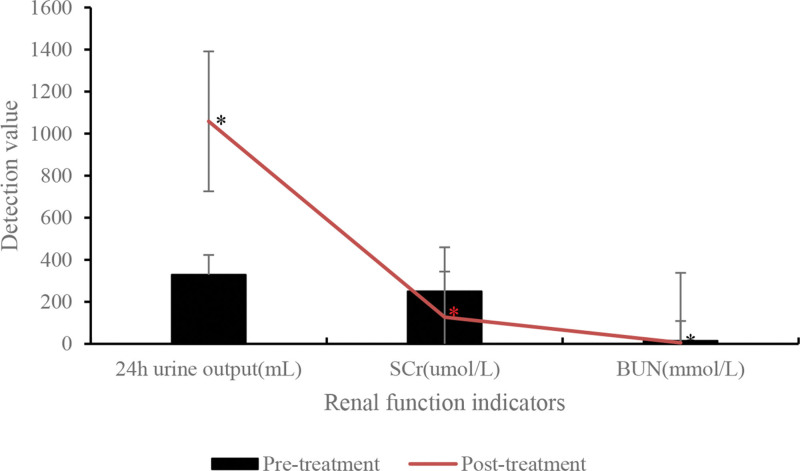
Comparison of renal function indicators. *P < .05 versus pretreatment. BUN = blood urea nitrogen, SCr = serum creatinine.

Renal function indicators before and after anti-inflammatory treatment in SA-AKI patients are presented in Table 12 and Figure 13. The results demonstrated a substantial increase in 24-hour urine output in SA-AKI patients after anti-inflammatory treatment versus pretreatment (*P* < .05). Furthermore, the levels of SCr and BUN in SA-AKI patients markedly decreased after treatment (*P* < .05).

**Table 12:** Comparison of renal function indicators.

Group	24-h urine volume (mL)	SCr (umol/L)	BUN (mmol/L)
Pretreatment	328.52 ± 97.54	249.52 ± 87.58	14.37 ± 4.73
Posttreatment	1058.42 ± 276.83*	126.74 ± 53.86*	5.08 ± 1.19*
*t*	7.583	5.736	5.648
*P*	<.05	<.05	<.05

BUN = blood urea nitrogen, SCr = serum creatinine.

* *P* < .05 versus pretreatment.

### 3.11. Analysis of anti-inflammatory treatment efficacy in patients

**Figure 14.**
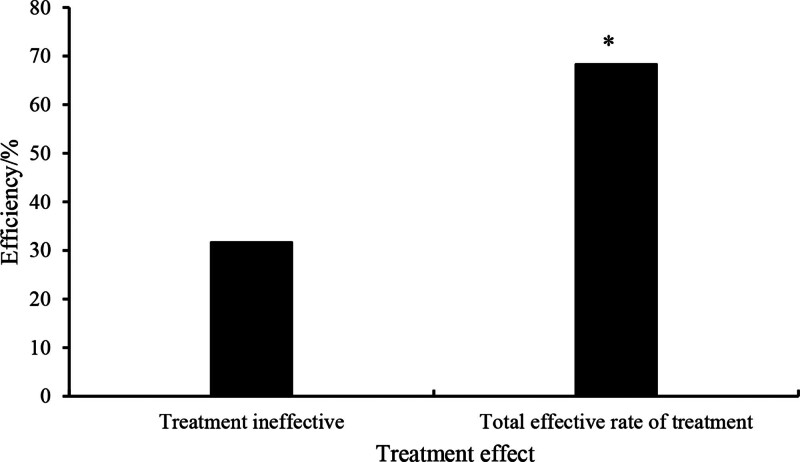
Analysis of efficacy in anti-inflammatory treatment among patients. *P < .05 versus patients with treatment ineffectiveness.

The results reveal that treatment with anti-inflammatory medication yielded a therapeutic effectiveness rate of 68.33% among patients, while 38.67% of patients exhibited treatment ineffectiveness. The utilization of anti-inflammatory medication significantly ameliorated disease symptoms in patients, with a markedly superior overall therapeutic efficacy rate compared to those for whom the treatment was ineffective (*P* < .05; Fig. 14).

## 4. Discussion

Currently, the internationally accepted diagnostic criteria for AKI mainly rely on changes in SCr, urine output, and glomerular filtration rate.^[29,30]^ Nevertheless, SCr as a diagnostic marker has certain limitations and shortcomings, leading to some limitations in the accurate diagnosis of AKI.^[31]^ These limitations pose a series of challenges to the prognosis and quality of life of AKI patients. Serum creatinine levels are influenced by various factors such as muscle mass, protein intake, and metabolic status. Hence, in some cases, even in the presence of renal impairment, SCr levels may still remain within the normal range, leading to delayed diagnosis.^[32]^ Additionally, SCr shows minimal changes in the early stages of AKI, resulting in lower sensitivity and specificity for early detection and intervention in patients. These limitations make the diagnosis based on SCr less accurate and reliable. In response to these issues, researchers have started to focus on finding new biomarkers to replace SCr and urine output, aiming to improve the diagnostic accuracy and early warning ability of AKI. These biomarkers may include cytokines in urine, markers of tubular injury, and metabolites in blood. By measuring their concentrations or changes in urine and blood, it is possible to more accurately assess the degree of kidney injury and its progression.^[33]^ Studies indicated that the mortality rate is extremely high in SA-AKI patients, and a considerable proportion of surviving patients require renal replacement therapy.^[34]^ This further emphasizes the importance of finding new diagnostic criteria. Timely and accurate diagnosis, as well as early intervention in AKI, can not only reduce the mortality rate and improve patient outcomes but also alleviate patient suffering and enhance their quality of life. Hence, there is a need to actively explore and evaluate new biomarkers as diagnostic indicators for AKI. Efforts to provide more accurate and early diagnostic methodologies and offer timely and personalized treatment plans for patients can improve the prognosis of those with AKI and ultimately enhance their quality of life.

NLRP, PCT, and HBP have been extensively studied to explore their diagnostic value in SA-AKI. NLRP is closely related to inflammation and immune regulation. Research has found that under stable conditions, NLRP1 inflammasome can exist in an autoinhibitory complex with dipeptidyl peptidases 8 and 9, which is activated by pathogen-encoded proteases upon infection. Additionally, the ORF45 protein of herpesviruses was found to activate human NLRP1 inflammasome in a non-protease-dependent manner.^[35]^ Among the NLRP-related proteins, NLRP3 inflammasome can activate the NF-κB pathway, promoting the transcription of pro-inflammatory cytokines or interleukins (IL-1β and IL-6).^[36]^ In a study on the diagnosis of late-onset sepsis in newborns, it was observed that the serum NLRP3 inflammasome in late-onset sepsis infants greatly increased versus control group. The optimal cutoff value for NLRP3 detection in diagnosing late-onset sepsis was > 3 ng/mL, with a sensitivity of 92.5% and specificity of 97.5%, suggesting that NLRP3 inflammasome could be utilized as a diagnostic biomarker for late-onset neonatal sepsis.^[37]^ This work also confirmed the association between NLRP and AKI. NLRP levels were greatly elevated in SA-AKI patients and positively correlated with the severity of renal injury. Conversely, NLRP levels in survival group were relatively lower. This suggests that NLRP may serve as a potential predictive marker for AKI. By measuring NLRP levels in the blood of SA-AKI patients, a more accurate assessment of the patient’s risk and prognosis can be made. Elevated levels of NLRP may indicate more severe renal injury and the need for more aggressive intervention and treatment. In AKI, the activation and regulation of the IR are crucial for the damage and repair of renal tissues. The elevation of NLRP may reflect the activation of the immune system, highlighting the significance of the IR in the development of SA-AKI.

Another biomarker associated with AKI is PCT, which is a precursor peptide substance.^[38]^ Under normal physiological conditions, PCT has low serum concentrations. Nevertheless, in cases of severe bacterial infection, fungal infection, sepsis, and multi-organ failure, PCT is released into the bloodstream, leading to a sharp increase in blood PCT levels.^[39]^ Hence, PCT is commonly utilized for the diagnosis of various infectious diseases.^[40]^ Studies have shown that PCT can reflect the severity of sepsis in patients and is a risk factor affecting patient prognosis and survival.^[41,42]^ A retrospective study involving intensive care unit-admitted patients utilized multivariate logistic regression, ROC analysis, and smooth curve fitting to evaluate the relationship between PCT levels and AKI, revealing that PCT can serve as a potential biomarker for AKI in female patients with bacterial septic shock under the age of 75.^[43]^ Chen et al suggested that the ratio of PCT to Alb was identified as an independent risk factor with robust and accurate risk assessment for adverse prognosis in sepsis-induced AKI patients. PCT levels were found to be closely related to the occurrence and severity of SA-AKI.^[44]^ Particularly in critically ill sepsis patients, high levels of PCT may indicate an increased risk of AKI.^[45]^ High levels of PCT are associated with poor prognosis and low survival rates. The sustained increase in PCT levels may indicate the ongoing presence of inflammatory reactions and further deterioration of kidney function. This work also confirmed the association between PCT and AKI. The research revealed that SA-AKI patients had significantly elevated serum PCT levels, which were positively correlated with the severity of kidney injury. In contrast, the NLRP levels in survival group were relatively lower. This suggests that NLRP may serve as a potential predictive indicator for AKI.

Serum HBP, a protein closely associated with IRs, has been found to hold predictive value in sepsis-related AKI.^[46]^ In sepsis-related AKI, when the body is exposed to infection or inflammatory stimuli, HBP is released into the bloodstream, leading to a notable increase in its concentration. A study involving 601 patients with severe sepsis or septic shock found that adding plasma HBP to the clinical risk model notably increased the AUC, indicating that plasma HBP added predictive value to known risk factors for SA-AKI.^[47]^ Another study of 794 patients with sepsis found that non-survivors had markedly higher serum HBP levels versus survivors, and it was confirmed that combining SOFA sepsis score with HBP could predict sepsis mortality.^[48]^ Similarly, it has been observed that SA-AKI patients exhibit greatly elevated HBP levels, which are positively correlated with disease severity. Furthermore, the level of HBP is also associated with the recovery of renal function and the prognosis of patients.^[48]^ This work also confirmed the association between HBP and AKI. It was found that SA-AKI patients had significantly elevated serum HBP levels, which were positively correlated with the severity of kidney injury. Hence, monitoring HBP levels can be utilized to assess the severity of sepsis-related AKI. Higher HBP levels are positively correlated with disease progression and deterioration, indicating an increase in intensity of IR and the extent of renal impairment. At the same time, HBP levels can serve as an important indicator for evaluating patient prognosis, with higher HBP levels being associated with poor renal function recovery and adverse outcomes.

Infection is a critical factor triggering the cascade of inflammatory reactions and leading to the occurrence of sepsis, with the majority of severe sepsis patients experiencing AKI.^[49]^ During this process, TNF-α plays a pivotal role as one of the key inflammatory mediators in the sepsis cascade.^[50]^ TNF-α promotes the release of itself and IL-1β and IL-6, through the activation of NF-κB signaling. This results in dysregulated immune system IR, leading to an uncontrolled immune-inflammatoryreaction. Researchers have found that ulinastatin can alleviate the IR by inhibiting neutrophils and IL-8 response.^[51]^ Yang et al noted that ulinastatin treatment remarkably reduced serum urea, creatine kinase, SCr, and myoglobin (Mb) levels, thereby alleviating the IR.^[52]^ In a septic rat model, ulinastatin was found to reduce the IR, inhibit macrophage autophagy, maintain VE-cadherin expression, and improve cortical and medullary perfusion.^[53]^ The results of this work demonstrated that anti-inflammatory treatment had a positive impact on the IR and clinical manifestations of SA-AKI patients, emphasizing the potential role of anti-inflammatory therapy in improving the prognosis of SA-AKI patients. The analysis showed that APACHE-II score and SOFA sepsis score are commonly utilized indicators to assess the severity and prognosis of patients.^[54,55]^ In SA-AKI patients, the APACHE-II score notably decreased, indicating that anti-inflammatory treatment can alleviate the severity of the disease and improve its prognosis. After anti-inflammatory drug treatment, the levels of inflammatory mediators also changed dramatically in SA-AKI patients. These inflammatory mediators include TNF-α, IL-1β, and IL-6, which play crucial roles in the IR. The results indicated that these inflammatory mediators decreased greatly after treatment, suggesting that anti-inflammatory therapy can effectively suppress the IR and reduce the release of related inflammatory mediators. IL-10 is an anti-inflammatory cytokine that plays a role in inhibiting the IR and regulating immune balance.^[56]^ In this work, it was observed that after anti-inflammatory treatment, the serum IL-10 level in SA-AKI patients increased substantially. This indicates that anti-inflammatory therapy can promote the release of the anti-inflammatory factor IL-10, thereby alleviating the intensity of the IR. Additionally, the levels of NLRP, PCT, and HBP in the serum greatly decreased after treatment. NLRP is an inflammasome-related protein, and its decrease may suggest a decline in inflammasome activity.^[57]^ PCT and HBP are infection markers, and their decrease may indicate a reduction in the severity of infection.^[58]^ These results further demonstrated the benefits of anti-inflammatory treatment for SA-AKI patients. Moreover, the notable reduction in serum levels of SCr and BUN suggested that anti-inflammatory treatment had a positive protective effect on renal function in SA-AKI patients. SCr and BUN are indicators utilized to assess kidney function, and their reduction indicates a lessening of kidney damage and improvement in renal function.^[59]^ In short, the results indicated that anti-inflammatory treatment significantly enhanced the clinical presentation and prognosis of patients with SA-AKI. There was a pronounced therapeutic effect observed in SA-AKI patients through this intervention. Suppression of IRs, modulation of inflammatory mediators, facilitation of anti-inflammatory cytokine release, and renal function preservation are likely to constitute the primary mechanisms underlying the efficacy of anti-inflammatory treatment.

The research findings revealed that relative to death group, SA-AKI patients in survival group showed slight decreases in indicators such as HR, diastolic blood pressure, systolic blood pressure, and blood oxygen saturation, but not considerable. The levels of blood creatinine, blood potassium, β2-MG, BUN, and Cys-C in survival group of SA-AKI patients were comparatively lower or higher than those in death group, with notable differences observed for β2-MG and Cys-C. Moreover, the serum levels of NLRP, PCT, and HBP in survival group of SA-AKI patients were dramatically lower. These biomarkers exhibited good predictive value for the severity of SA-AKI. In terms of patient scores on the APACHE-II and SOFA sepsis scores, the APACHE-II score and SOFA sepsis score of SA-AKI patients in survival group were notably inferior to those in death group. This indicates that both of these scoring systems can help predict the prognosis of SA-AKI patients. The efficacy analysis of NLRP, PCT, and HBP in diagnosing the severity of SA-AKI was conducted by plotting ROC curves, and the AUC for NLRP, PCT, and HBP was found to be 0.783, 0.847, and 0.803, respectively, with substantial differences. This suggests that these indicators have predictive value in diagnosing the severity of SA-AKI. In conclusion, based on these findings, it can be inferred that the serum levels of NLRP, PCT, and HBP may be associated with the severity and prognosis of SA-AKI. Nevertheless, further clinical studies are needed to validate these results and determine the therapeutic effects of anti-inflammatory drugs in the context of sepsis-related AKI.

Several limitations and potential sources of bias in this study should be acknowledged. First, this was a retrospective, single-center observational study, which may have introduced selection bias. Only hospitalized patients diagnosed with SA-AKI at our institution were included, and therefore the study population may not be fully representative of all SA-AKI patients in different clinical settings. Second, confounding bias cannot be completely excluded. Although all patients received standardized sepsis management, including fluid resuscitation, antimicrobial therapy, and supportive care, unmeasured confounding factors such as differences in baseline disease severity, timing of intervention, infection source, and concomitant medications may have influenced clinical outcomes and biomarker levels. While multivariate logistic regression analysis was performed to adjust for potential confounders, residual confounding remains unavoidable due to the observational nature of the study. Third, information bias may exist because clinical and laboratory data were collected from electronic medical records, which may be subject to missing or inaccurate documentation. Finally, the absence of a control group without anti-inflammatory treatment limits causal interpretation of the therapeutic effects of ulinastatin. The observed improvements after treatment should therefore be interpreted as associations rather than definitive evidence of efficacy. Future prospective, multicenter, randomized controlled studies with appropriate control groups are warranted to validate these findings.

## 5. Conclusion

This work demonstrated the potential value of NLRP, PCT, and HBP as diagnostic biomarkers in predicting sepsis-related AKI. By comparing SA-AKI patients in survival group and death group, it was found that the levels of NLRP, PCT, and HBP were lower in survival group. Hence, these indicators can serve as auxiliary tools for early prediction and clinical assessment of SA-AKI. NLRP, PCT, and HBP also exhibit high accuracy in diagnosing the severity of SA-AKI. ROC curve analysis indicates that they have high AUC values in predicting SA-AKI diagnosis, further supporting their potential utility as complementary markers for assessing the severity of SA-AKI. In conclusion, this work highlights the significance of NLRP, PCT, and HBP as diagnostic biomarkers, along with the importance of APACHE-II and SOFA scores as clinical assessment tools in sepsis-related AKI. Furthermore, anti-inflammatory therapy effectively reduces inflammation levels, improves kidney function, increases urine output, and alleviates renal damage in SA-AKI patients. Nevertheless, this work is purely observational, and further large-scale, randomized controlled clinical trials are needed to validate these findings and explore additional effective approaches for treating SA-AKI. Investigating their specific roles in treatment planning and mechanism research will contribute to enhancing the diagnosis and treatment strategies of sepsis-related AKI and ultimately improve patient prognosis and survival rates.

## Author contributions

**Conceptualization:** Zhidong Fang, Shuqin Cui, Xian Liu, Min Shi, Wei Zheng, Jing Xue, Li Chen.

**Data curation:** Zhidong Fang, Shuqin Cui, Xian Liu, Min Shi, Wei Zheng, Jing Xue, Li Chen.

**Formal analysis:** Zhidong Fang, Shuqin Cui, Xian Liu, Min Shi, Wei Zheng, Jing Xue, Li Chen.

**Funding acquisition:** Zhidong Fang, Shuqin Cui, Wei Zheng.

**Investigation:** Shuqin Cui.

**Writing** – **original draft:** Zhidong Fang, Shuqin Cui.

**Writing** – **review & editing:** Zhidong Fang, Shuqin Cui.
